# A Study of the Literature of Puerperal Eclampsia

**Published:** 1877-03

**Authors:** Smith Baker

**Affiliations:** Whitesboro’, N. Y.


### A STUDY OF THE LITERATURE OF PUERPERAL ECLAMPSIA.

By SMITH BAKER, M.D., Whitesboro', N. Y.

As early as the beginning of this century, a Dr. Hamilton is reported to have noticed that puerperal eclampsia was often preceded by anasarca; but this observation does not seem to have impressed the profession as having much importance, for in 1829, Dr. Harrison is recorded in the *London Lancet*, as believing the disorder due to " injury inflicted upon the uterine nerves." " By the too rapid and forcible dilatation of the cervix uteri, os tincæ, or vagina, some of the nervous fibrils are so suddenly elongated as to become fretted, unduly stretched, or, perhaps, actually torn, if not burst asunder."

About the same time Dr. Blundell, in the same periodical, said, in speaking of treatment, " a main remedy is the abstraction of blood, as largely as the patient may safely bear. There is a second remedy of very great importance, viz., the thorough evacuation of the alimentary canal. * * You generally, if not always, find symptoms of cerebral efflux of blood; hence, the importance of the complete refrigeration of the head. Lastly, deliver, if you can do it gently, if not, leave to nature." He gave two cases illustrative of his opinions, one of which lived and the other died.

In 1844 the editor of the *London Lancet* quoted several authors as representatives of the opinions of that time concerning the etiology and pathology of this affection. According to Ramsbotham, the most usual proximate cause is probably pressure on the brain, produced by the rupture of a vessel, by serous exudation into the ventricles, or by simple congestion of the cerebral vessels themselves, the affection originating most commonly in some deranged state of the uterus itself, an irritation being propagated from that organ to the brain. According to Dr. Rigby, the exciting cause "is the irritation arising from the presence of the child in the uterus or passages, or from a state of irritation thus produced continuing after labor," while the predisposing causes are "pressure on the abdominal aorta, uterine contraction, constipation, retention of urine, injuries to the head, cerebral disease and mental excitement." According to Burns, the cause is uterine contraction, and sometimes "a neglected state of the bowels." "The sympathetic irritation is almost invariably accompanied by an affection of the vascular system, productive of great determination to the head * * which aggravates the evil, and, indeed, becomes the chief source of danger." "After death, we sometimes find turgescence of the vessels of the brain, but very often no mark of disease elsewhere. According to Lee, "the phenomena are due to a condition produced by sympathy with the nervous system of the uterus." According to Locock, the immediate causes are often very obscure. A loaded state of the vessels of the brain appears to be one. At times the brain seems influenced by distant irritation in the uterus or digestive organs. Attacks are due to a loaded or disordered stomach, or by indigestible food, the straining of labor pains, and even the disturbance of rush of blood to the head, caused by the earlier uterine contractions, will sometimes bring on the convulsions.

In the same issue of the *Lancet*, W. Tyler Smith attempted to demonstrate that puerperal convulsions are a purely "excito-motor phenomenon." "The convulsions commonly occur," he says, "when the spinal marrow has been acted upon by an excited condition of its incident nerves coming from the uterine organs, such excitement depending on labor, pregnancy, or the puerperal state. While the spinal marrow remains under the influence of these stimuli, convulsions may arise from two series of causes: those acting primarily on the spinal marrow, or *centric causes*, as, 1, loss of blood; 2, pressure from congestion, effusion, etc.; 3, asphyxia from closure of the glottis; 4, emotion; and those affecting the extremities of the incident nerves, or *eccentric causes*, as, 1, irritation of uterine incident nerves; 2, irritation of incident spinal nerves of the rectum; 3, irritation of incident gastric fibres of the pneumo-gastric nerve; 4, irritation of the vesical branches and of those of the surface of the body."

A year later the same writer said, in the same periodical, of the treatment of this affection, that "blood-letting, in fullness of the vascular system, is the most powerful sedative of spinal action that we possess, and is the grand remedy in the simpler forms of convulsion, where the disease depends on stimulation of the spinal marrow by excess of blood. But when the circulation is reduced considerably below par, loss of blood becomes an actual stimulant to the spinal marrow. In convulsions occurring in delicate anæmic women, bleeding is generally inadmissible, being, in fact, itself an exciting cause of the disease. In intermediate conditions, venesection should be followed by stimulation." In addition, "cold water to the head and face, in order to open the glottis, and to relieve cerebral congestion, and avoidance of all emotional excitement," seemed to him of importance.

In 1849 Dr. Murphy gave, in the *Medical Gazette*, as the predisposing causes of this affection, "hyperæmia, anæmia, impure blood;" while he considered the proximate causes to be "morbid irritation of the uterus; morbid irritation of other organs."

In 1855 a case was reported in the *Lancet* by Dr. Winn, treated by abstracting "twelve ounces of blood and giving a few small doses of calomel." Recovery followed. A year after, however, Dr. Radford reported a case, the treatment of which was "bleeding to twenty ounces, purging with croton oil, and bleeding again profusely," because the convulsions still continued; that terminated fatally.

In 1856 W. Tyler Smith, in the *Lancet*, reiterated his account of causes given ten years previously, and said of bleeding, that in plethoric states "it is in this disease *curative* in its action on the spinal marrow; *preventative* in its action on the brain." He also directed that we "should look to the albuminuria," and to allay the uterine irritation we "may let off the liquor amnii."

To Prof. J. Y. Simpson is credit generally given for having first called attention to the fact that albumen is sometimes found in the urine of pregnant women; while to Dr. John C. W. Lever a like honor is given for having reported, in 1843, that in nine cases out of ten albuminous urine accompanied puerperal eclampsia. But up to about twenty years since this fact does not seem to have commanded the attention of practical physicians to any extent. With the impetus of such a name as Tyler Smith, it is not strange that the profession afterterward manifested a deep interest in the subject, and heeded his injunction to "look to the albuminuria."

Contemporaneously with this new phase in the etiology of this disease was the introduction of chloroform as a therapeutic agent. In the *Lancet* of October 5, 1859 a case is reported by Dr. Bullen, treated by chloroform inhalation with recovery. But his action may have been based upon the comprehensive propositions of Dr. Carl Braun, published in the same year at Edinburg. Some of them were as follows:

"Convulsions in females during the generative period depend either upon hysteria, epilepsy, disease of the brain, mineral or vegetable poisoning, or upon uræmic intoxication. The most frequent cause of eclampsia is uræmia and Bright's disease.

"In cases of Bright's disease during pregnancy, if carbonate of ammonia be found in the blood, a speedy outbreak of convulsions may be expected. Eclampsia, *i. e.*, uræmic convulsions, has no immediate connection with the pains and process of labor. Albumen will be found in all cases of eclampsia not dependent upon hysteria, epilepsy, primary cerebral disease, or poisoning. The injurious effects of bleeding in eclampsia have been observed by Kiwisch, Litzman, Sedgwick, Blot and King, and the uncertainty of the practice has been confirmed by our own experience. Chloroform inhalations are the best means of mitigating and bringing to an end uræmic convulsions either during pregnancy labor, or the puerperal period. The diuretics most to be relied upon for the relief of uræmia and Bright's disease are the benzoic, citric and tartaric acids. Artificial delivery is not, as a rule, to be resorted to in Bright's disease, but it may be in actual uræmic convulsions. The most appropriate method of producing labor is by forcibly dilating the vagina [os uteri?] by means of a caoutchouc apparatus.

In the *Amer. Jour. Med. Sci.*, July, 1860, Dr. Chas. A. Lee reported a case treated successfully by chloroform, and also said that congestion of the kidneys from mechanical pressure must be the cause of the albuminous urine, inasmuch as this condition is met with most frequently in primiparæ, and disappears in two or three days at farthest after parturition. He believed that chloroform, in the vast majority of cases, "is the sheet anchor of our reliance."

In 1863 Rosenstein expressed his belief that puerperal convulsions are attendant upon an "alteration of the circulation within the brain." Pressure and diluted serum occasion "œdema and secondary anæmia of the brain, and thus may call forth convulsions."

In the *Medical Times and Gazette*, of July 4th, of the same year, strong testimony to the efficiency of chloroform in controlling puerperal convulsions, is borne by Dr. Murphy and Dr. Braxton Hicks; but three years later, an abstract of a report on this affection, by Dr. W. C. Hall, to the Ohio State Medical Society, published in the *Amer. Jour. Med. Sciences*, sums up by declaring that the nature of puerperal convulsions is yet undecided, the causes undetermined; that uræmia, as the only cause, is denied, if not disproved; that *post-mortem* examinations are unsatisfactory. The almost universal testimony is found in favor of reasonable depletion; the use of chloroform and opiates is strongly recommended, and almost as strongly denounced. "Speedy delivery is the only thing universally agreed upon."

In Vol. 8, *Trans. London Obstet. Society*, Dr. Braxton Hicks says that uræmic eclampsia causes nephritis; the nephritis and the convulsions are produced by the same cause, *e. g.*, some detrimental agent in the blood, irritating both the cerebro-spinal system and other organs at the same time; or the highly congested state of the nervous system, as is produced by the spasm of the glottis in eclampsia, is able to produce kidney complication.

In 1868 Dr. G. Swayne, in the *British Medical Journal*, regrets the disuse of the lancet, and objects to chloroform as a substitute for it.

In the *Michigan Univ. Med. Jour.*, for 1870, Prof. H. S. Cheever reported a case treated by evacuating the stomach, abstracting fifteen ounces of blood, giving five grains of calomel and jalap, each, supplemented in due time by an injection, to secure catharsis, sponging the surface with warm vinegar, and giving small doses of compound jalap powder, to secure action of the skin and kidneys, and keeping the patient well under the influence of chloroform throughout. At the end of twenty hours the child and placenta were successfully delivered, and after much persuasion the former made to live. The chloroform was now withdrawn, but in two and one-half hours the mother had another convulsion, and repeatedly had them, notwithstanding the chloroform was again resumed. Iodide of of potassium, strong beef tea, etc., were given until the second day after, when they ceased. Sixty hours later, although injections of morphia, brandy and beef tea were given, the patient died. Albumen was found in the urine throughout.

In the *Transactions of the N. Y. State Med. Society* for 1871, Dr. Harvey Jewett has an article. He had this experience: "Prior to 1860 I find ten recorded cases that were treated by copious and repeated bleeding, blisters, sinapisms, cold, opium, etc. Out of the ten cases thus treated, seven died. Since that period I find six cases that were treated with chloroform and drastic cathartics, not one of the number were bled, and all recovered."

In *Guy's Hosp. Reports* for 1870–'71, Dr. J. J. Philips says that "the present diminished mortality is probably chiefly due to the less free depletion that is now practiced; and the chief reliance should be placed on chloroform, which prevents the recurrence or diminishes the violence of the paroxysms."

In 1870, Dr. R. D. Fox, in the *Lancet*, reported a case of a fifteen and one-half year old girl who, continuing to have convulsions for hours after delivery, was given a half drachm dose of this agent with marked success.

Dr. Robert Barnes, in his Lumleian lectures (1873) takes this position: Pregnancy and labor require an extraordinary supply of nerve force. This implies a corresponding organic development of the spinal cord. The provision of such nerve force implies a greatly augmented irritability of the nervous centres, rendering them more susceptible to emotional and peripheral impressions. The disturbances in nutrition, occasioned by pregnancy, almost always entail some alteration of the blood, which increases the irritability of the nervous centres. When the blood change is marked by albuminuria, a poisonous action of peculiar intensity is exerted upon the nervous centres, tending to produce eclampsia. Rational treatment must accord with the two great factors in the production of such diseases, namely, exalted nervous irritability and lowered or empoisoned conditions of the blood.

His plan of treatment embraces the induction of labor, where practicable, especially by "puncturing the membranes and leaving the rest to nature;" the induction of anæsthesia by chloroform, which proceeding "now rests upon a solid foundation of clinical facts;" and "when the patient can swallow in the intervals of the fits, the giving at first a scruple or half-drachm dose of chloral, and then every three or four hours a like quantity of bromide of potassium or ammonium." He also says: "I am one of those who think there is more of fashion than of wisdom in the almost absolute disuse of the lancet; but in this particular case I do not regret the disuse into which it is falling." He would cut off all emotional excitants, and says, "there is no fact in medicine of which a stronger conviction has been forced upon me by observation than this: that all peripheral irritation (*i. e.*, blisters, mustard, poultices, cold, etc.,) is injurious in eclampsia. Hence the rule, where the situation dictates manipulation of any kind, to lull the system in the artificial sleep of anæsthesia."

During the same year (1873), Dr. John Barclay, in the *British Medical Journal*, mentions giving, in a severe case, as a sort of "forlorn hope," half a drachm of chloral in a small starch enema, with results entirely satisfactory, as well as surprising. Dr. Allan D. Barclay, in the same Journal, reports a successively "slight, bad and worse" case checked by the same means.

Dr. T. More Madden, of Dublin, in 1874, (*Medical Press and Circular*,) declares that "some advance has recently been made in the prophylaxis and management of this disease, but its causes still remain *subjudice*, sharing among a variety of circumstances." The state of the uterus during gestation, the remarkable condition of nervous susceptibility, the interference with the venal function, the cerebro-spinal congestion, the irritation of the same by the circulation of vitiated blood, producing a direct toxic effect, all combine to produce such a hyperæsthetic or irritable condition of the excito-motor nerve substance as to "need only the addition of the uterine irritation * * to cause the pent-up nerve force to burst into uncontrollable action," producing eclampsia. He distinguishes puerperal eclampsia from similar affections, by observing that the former is "preceded by œdema of the upper extremities, face and eyelids, pain in the lumbar region, and albuminuria; that for some days before the attack the patient generally complains of *malaise* followed by headache, giddiness, confusion of thought, or peculiar irritability of temper." Treatment with him is especially prophylactic, and has reference to relieving the kidneys by cupping and fomentations over the loins, the free use of diluents, and the continuous administration of mild diuretics, notably, "colchicum in small and guarded doses; in assisting nature to purify the blood by saline cathartics and diaphoretics, and soothing nervous irritability by sedatives, especially bromide of potassium and belladonna."

In the *Amer. Jour. of Obstetrics*, for August of the present year (1876,) Dr. Henry F. Campbell, of Georgia, puts it "that the proximate cause of eclampsia having been recognized as centric and peripheral irritation, or exaggeration of reflex excitability, then the sole, the grand and consummate indication is to quiet and subdue irritation. * * * The well-known and universally acknowledged superiority and efficiency of opium and its preparations for controlling irritation renders it the first, the most ready, and the most promptly effectual of all the means at the command of the practitioner. * * * Next to opium, in its general applicability, and superior to opium in many specific cases, utterly indispensable to some, we should rank blood-letting as *the* sedative;" and he claims that if not resorted to so frequently as formerly, venesection should at least be retained as "one of the most reliable of all our reliances, while chloroform, chloral, bromides and quinia may all be considered with this as possessing one common therapeutic endowment—power to subdue nervous irritation."

In addition to these selections from various periodicals, we glean from the text-books of the present day the following opinions: Says Cazeaux (1870), "It is evident that all these (the causes) have a tendency to produce irritation of the nervous centres. This irritation is direct, when due to the immediate contact of vitiated blood, and indirect, or by reflex action when it follows the excitement of a distant organ, as the bladder, uterus, etc. I am happy to find, in the work by Scanzoni, a confirmation of these views. * * * Since albumen is present in the vast majority of eclamptic women, it, or the disease of which is a symptom, may be rightfully regarded as the predisposing cause of eclamptic convulsions. I say the only predisposing cause. Usually, general venesection will first be preferred. The vein should be opened largely, and the blood should flow in a full stream. Should it dribble away, or the jet be very small, the bleeding is almost useless, and another vein had better be opened at once. Simultaneously it is advisable to produce a derivature to the intestinal canal and skin." * * "When eclampsia comes on during either pregnancy or labor, and the closure or undilatibility of the cervix makes it impossible to effect delivery, or when the attacks, having resisted bleeding, and revulsions are very frequent, and threaten the life of both mother, and child, the use of chloroform may be of some service."

Says Elliot (*Obst. Clinic*, New York, 1873,) "the greatest advantage gained from our knowledge (recent) has been in prophylaxis. * * Instead of suspecting the brain, we now study the kidneys. That we have reached the *ultima thule* of our investigations is not even imagined. * * The pathology of the blood remains comparatively unexplored, and the relation of that fluid, and of the nervous system itself, to the proximate cause of the convulsions, as well as the influence of other toxæmic conditions, offer wide fields for exploration. Pregnancy presents the great clinical peculiarities of being a special excitor, in very many cases, of albuminuria; of materially developing morbid conditions in many chronic cases in which they might possibly have remained latent for a much longer period. Albuminuria, in pregnancy * * entails a special liability to some dangers, such as convulsions and mania."

"Prophylaxis of puerperal eclampsia — Eliminate through the bowels, the skin and perhaps the kidneys. Consider the advisability of inducing labor and of abstracting blood. Diminish the supply of meat and undigestible food. Remove exciting and depressing influences. Do not debilitate the patient. Ward off threatening attacks with chloroform or sedatives. I see every reason for believing that chloroform is the most prompt and certain agent that we possess for moderating the violence and preventing the recurrence of the convulsions."

Schrœder (1873,) quotes Halbertstina as believing that the convulsions are caused by the retention of the constituents of the urine, and that this retention is due "to pressure of the uterus on the ureters." While according to the same author, Traube believes "that the loss of albumen, and the consequent hydræmia cause by the simultaneous hypertrophy of the left ventricle, a greater pressure in the arterial system, and this leads to œdema of the brain, which shows itself as coma when the cerebrum is œdematous, or as convulsions when the middle portions are affected. According to this theory, an increased pressure suddenly produced in the aortic system, in cases when the blood is in a high degree of hydræmia, leads to hyperæmia of the brain; but on account of the watery condition of the blood, œdema of the brain is the necessary consequence of this hyperæmia. The passage of water into the tissues, again, exerts a mechanical pressure upon the blood-vessels, and leads consecutively to anæmia of the brain. The effect of this acute cerebral anæmia is observed as an epileptic seizure."

He considers the treatment to be effectual only "when narcosis is absolute; that is, when the patient is quite unconscious, so that the voluntary muscles no longer contract. As long as the eyelid quivers, or the body is in any way thrown about, another dose is requisite." This narcosis he would accomplish by means of chloroform, or by the hypodermic injection of morphia. He quotes Reickhard as authority for the sub-cutaneous injection of chloral, and Martin as testifying to the efficiency of the same agent given as an enema.

Dr. Fordyce Barker (Puerperal Diseases, 1874,) refers to the work of Frankenhaueser, of Jena, "On the Nerves of the Uterus," in which he says "is demonstrated a direct communication between the nerves of the uterus and the renal ganglia. He (Frankenhaueser) believes that the sudden occurrence of eclamptic attacks following all external sources of irritation (as pressure of fœtal head upon cervix, digital examinations, etc.,) and from emotional causes, goes to prove that the nervous system, and not the vascular system, is the starting point of puerperal convulsions."

In the same connection, Dr. Barker himself considers that "clinical observations have established that the following conditions are predisposing causes of eclampsia in puerperal women, viz.: albuminuria, hyperæmia, anæmia, uræmia, primiparity, and, perhaps, atmospheric influence, and that anything which produces direct or indirect irritation of any part of the nervous system may bring on convulsions, as in the pregnant, indigestion, constipation, retention of urine, excessive distention of the uterus, reflex pains, or moral shocks. During labor, besides all these causes, anything which makes pain severe."

Treatment with Dr. Barker is prophylactic, as "removing renal and local congestions, curing anæmia, inducing labor, if necessary, using chloroform, permitting the patient to lose blood, watching the renal secretion, relieving the bladder, and tranquilizing, by means of opiates, or otherwise, as when the attack occurs before labor. If the pulse be strong and hard, with great fullness of the vascular system, and the appearance of the face indicates cerebral congestion, bleed at once. The bleeding is sedative to nervous irritation, it removes the tension from the brain, and protects it from injury." He then gives a brisk purgative, administers chloroform by inhalation, and also a hypodermic injection of morphia. He has been disappointed with chloral.

After this somewhat extended reference to the conclusions of others respecting the etiology and pathology of puerperal eclampsia, it may appear almost presumption to add any suggestion or experience of my own.

It may not seem a very great ascent from the "actually torn or fretted" uterine nerve of nearly half a century since to the "exaggerated reflex excitability," or direct anatomical connection between the "uterine nerves and renal ganglia of to-day;" nor from the "more bleeding the better" doctrine of the past to the "special venesection, chloroform, or eliminato-sedative treatment of the present." But a great advance is, indeed, evident, when we consider that during this time the profession has recovered from the paralysis of routinism to the more healthy condition which permits circumstances to intimate, and reason to dictate, the course to be elected and pursued in such given case; while, also, at the same time, the amount of suffering and the rate of mortality have been largely reduced.

What is needed in addition, just now, is a sharply-defined summary of what needs clearing up, by the study of morbid anatomy and pathology, and of our present knowledge of the means of prophylaxis and treatment.

With reference to morbid anatomy in this disease, it seems desirable to investigate 1, whether there is an actual increase in the structural elements of the spinal cord; 2, whether there is a direct communication between the nerves of the uterus and the renal ganglion; 3, whether there is an unusual condition of the uterine walls or intra-uterine elements; 4, whether there are actual lesions of the kidneys and bladder.

The study of pathology should include the following points: 1, the actual condition and relative proportion of the blood constituents; 2, the actual condition of the various secretions; 3, the question of exaggerated reflex excitability, having special reference to the relation of the blood to the brain and spinal cord; 4, the special influence of the puerperal state; 5, the special influence of the atmosphere and other external conditions.

Although this comprises a wide field for exploration, the combined efforts of the profession may soon accomplish the desired result, if each case occurring in private or hospital practice be accurately observed and faithfully reported. Until then we may act safely, perhaps, in adopting the following plan of treatment:

At a preliminary examination, to be held from two to four weeks before the expected labor, determine so far as possible, whether there be, *a*, hyperæmia; if so, prescribe a low diet, saline cathartics, and potassic bromide thrice daily; *b*, if there be anæmia, order a good diet, tr. ferri chlor. after meals, and an aperient pill at bedtime, if necessary; *c*, if œdema or albuminuria exist, these will need the exhibition of tr. ferri chlor. with meals, and potassic iodide between meals and at bedtime; *d*, headache, malaise, irritability, etc.; for these, remove the cause, if possible, and if necessary give sufficient opiate to effect relief.

During labor, if convulsions occur, seek out the special conditions of the patient and act as indicated. A full, strong, bounding pulse, and a bright colored mucous membrane will require venesection carried to the extent of securing its sedative effect, a half-drachm dose of chloral per mouth or rectum, means for inducing a movement of the bowels, and a free perspiration, relief of the bladder, if distended, and the exhibition of chloroform for anticipating and controlling the convulsions. A feebler pulse, pale membranes, and œdema will, as a rule, require or permit an active cathartic only, and chloral and chloroform. If the convulsions recur, (unless it be previous to the seventh month, when repeated hypodermic injections of morphine should be given, and the labor left to nature for a longer time,) proceed quickly, but gently, to dilate the os uteri by means of tents and Barnes' dilators, puncture the membranes, excite uterine contractions by means of warm uterine douche and ergot, and deliver as soon as possible.

After the delivery is accomplished, let the uterine hæmorrhage proceed moderately, inject, hypodermically, one-sixth to one-quarter grain of morphia, and give broths and stimulants as required; exercise extreme care about the after attentions, as bathing, etc., and prohibit and prevent all disturbances by friends, or otherwise.

In fine, by food, tonics and stimulants, by purging, bleeding or sweating, by quietness, or delivering, by opium, bromides, chloral and chloroform, each in its time to suit the *peculiar circumstances of individual cases*, and guided by judgment unobscured by theories, we may be more successful than hitherto at the bedside of our patients with puerperal eclampsia.

